# Social network-based ethical analysis of COVID-19 vaccine supply policy in three Central Asian countries

**DOI:** 10.21203/rs.3.rs-745691/v1

**Published:** 2021-07-26

**Authors:** Timur Aripov, Daniel Wikler, Damin Asadov, Zhangir Tulekov, Totugul Murzabekova, Kerim Munir

**Affiliations:** Tashkent Institute Of Postgraduate Medical Education; Harvard University; Tashkent Institute Of Postgraduate Medical Education; Al-Farabi Kazakh National University; Kyrgyz-Netherlands Community of Volunteers Tuberculosis Foundation; Boston Children’s Hospital

**Keywords:** COVID-19, vaccine, bioethics, low- and middle-income countries, Central Asia

## Abstract

**Background:**

In the pandemic time, many low- and middle-income countries are experiencing restricted access to COVID-19 vaccines. An access to imported vaccines or ways to produce them locally becomes the principal source of hope. But developing a strategy for success in obtaining and allocating vaccines is not easy task. The governments in those countries have faced difficult decision whether to accept or reject offers of vaccine diplomacy, weighing price and availability of COVID-19 vaccines against concerns over their efficacy and safety. Our aim was to analyze public opinion regarding the governmental strategies to obtain COVID-19 vaccines in three Central Asian countries, focusing particularly on possible ethical issues.

**Methods:**

We searched opinions expressed either in Russian or in the respective national languages. We provided data of the debate within three countries, drawn from social media postings and other sources. The opinion data was not restricted by source and time. This allowed to collect a wide range of possible opinions that could be expressed regarding COVID-19 vaccine supply and public’s participation in vaccine trials. We recognized ethical issues and possible questions concerning different ethical frameworks. We also considered additional information or scientific data, in the process of reasoning.

**Results:**

As a result, public views on their respective government policies on COVID-19 vaccine supply ranged from strongly negative to slightly positive. We extracted most important issues from public debates, for our analysis. The first issue involved trade-offs between quantity, speed, price, freedom, efficacy and safety in the vaccines. The second set of issues arouse in connection with the request to site a randomized trial in one of countries (Uzbekistan). After considering additional evidences, we weighed individual with public risks and benefits to make specffic judgements concerning every issue.

**Conclusions:**

We believe that our analysis would be a helpful example of solving ethical issues that can rise concerning COVID-19 vaccine supply round the world. The public view can be highly critical, helping to spot such issues. An ignoring this view can lead to major problems, which in turn, can become a serious obstacle for the vaccine coverage and epidemics’ control in the countries and regions.

## Background

For many low- and middle-income countries (LMICs), equitable access to COVID-19 vaccines becomes essential for avoiding catastrophic loss of life, health, and economic wellbeing in the current pandemic. According to the Eurasia Group report, the COVID-19 vaccines will generate economic benefits up to 466 billion US dollars by 2025 among the ten major economies [1]. The pandemic has caused huge devastation even in some of the wealthy countries ranked highest for epidemics preparedness. For the LMICs, the emergency presents even greater burden, as they do not have resilient public health systems, including limited capacity for intensive care, and cannot afford to maintain living standards through government payments.

With the fast-moving pandemic, the COVID-19 Vaccine Global Access (COVAX) initiative has been the best hope for worldwide immunization against the coronavirus in LMICs. A herd immunity to stop COVID-19 epidemic is evaluated approximately at 23 (67%), assuming a reproduction number (*R*_*0*_) estimate in 3 [2, 3]. This rate is much lower than were for measles, polio and smallpox. However, the situation was not so gracious as anticipated. Any priority-setting rule for allocating vaccines among LMICs would invite ethical debate, and this has been the case for COVAX’s, which tracks population size over other indicators of acute need [4, 5]. But the importance of this ethical choice for LMICs is overshadowed by the serious shortfall of vaccines available to COVAX for distribution. Countries producing vaccines, including those supporting COVAX, have practiced “vaccine nationalism”, directing available doses to domestic needs for all citizens before any consideration of exporting to foreign nationals who may be in more acute peril.

In such restrictions, each of these LMICs must develop a strategy for obtaining and allocation of COVID-19 vaccines to avoid disaster. Understandably, the governments of LMICs and world pharmaceutical companies were interested in direct contacts to supply their communities with the necessary doses of the vaccine. This could guarantee faster profit for vaccine producers and less vaccination cost for recipient countries, leading higher overall public health benefits [6]. Several LMICs have received their first vaccine shipments not from COVAX and world pharmaceutical companies, but from China and Russia, countries that use the vaccines to expand their influence, while the US has stood on the sidelines [7]. India could also be considered an important contributor in vaccine distribution due to its massive manufacturing capacity. However, as a response to severe epidemic with raw material shortages, this country stopped its vaccines exports and started importing them [8]. The Pfizer/BioNTech vaccine, first to demonstrate 95% efficacy in a large multinational trial [9] and approved in the US and the EU for mass vaccination has been hindered for distribution to limited manufacturing capacity. Although China has withheld claiming high efficacy of its Sinovac or Sinopharm vaccines until the end of phase III trials, Russia had declared its Sputnik V vaccine at 95% efficacy based on preliminary results [10]. The country reported phase I-II trials results last among leading producers; however, registered it first for emergency use. Both China and Russia have actively offered early and low-cost supply of their vaccines round the world, sometimes proposing that recipient countries furnish a human subject site for randomized controlled vaccine trials. The reason, in addition to economic and political ones, has been the need to garner data about long-term safety. Although these sites have included highly populated countries such as Brazil, they have also included neighboring countries, such as Uzbekistan. The governments in those countries have faced difficult decision whether to accept or reject offers of vaccine diplomacy, weighing price and availability of COVID-19 vaccines against concerns over their efficacy and safety.

The primary objective of our study was to analyze public opinion regarding the governmental strategies to obtain COVID-19 vaccines in three former-Soviet Union Central Asian countries: Uzbekistan, Kyrgyz Republic (Kyrgyzstan) and Kazakhstan. Public participation in these policy choices – the voice of those who will be directly affected by the outcomes – contributes to the ethical soundness of the resulting choices [11].

## Methods

Data was collected on range of opinions that have been expressed by members of the public as well as politicians, officials, and experts as reported in webpages of leading newspapers, TV channels and social media outlets. The opinion data was not restricted by source and time. This allowed to collect a wide range of possible opinions that could be expressed regarding COVID-19 vaccine supply and public’s participation in vaccine trials. The initial search for these data from our three countries relied on the Google search engine. This was supplemented with data from Facebook pages of news media, which are popular in three countries, such as Radio Liberty, Gazeta.uz, Sputnik and others.

The Russian equivalents of the following English keywords and their combinations were used for the search: *“COVID-19, vaccine, Uzbekistan”; “COVID-19, vaccine, Kazakhstan”*and *“COVID-19, vaccine, Kyrgyzstan”.* We searched opinions expressed either in Russian or in the respective national languages (Uzbek, Kazakh and Kyrgyz). We used meaning-based (abstractive) approach to summarize public opinions on vaccine supply and participation in vaccine trials. This helped to consider every view from different sources with different language use [12].

### Inclusion criteria

We decided to include postings from people living in Uzbekistan, Kyrgyz Republic and Kazakhstan at the time of posting. This could contribute more to engaging voices of those who are directly affected by the policy of vaccine supply in their countries. Because of global travel restrictions, few of these postings originated from outside the boundaries of our countries. The postings included were not restricted by ethnicity, residency, age, sex and social status of their authors.

### Ethical analysis

We sought debate over issues of COVID-19 vaccine supply, including participation in the vaccine trials in the Central Asian LMICs, that offered ethical views and reasoning, as opposed to disputes over theology, ideology, or law. At the beginning of our analysis, we recognized ethical issues and possible questions concerning different ethical frameworks [13, 14]. We considered additional information or scientific data, in the process of reasoning. Examples of internationally-accepted ethical standards included the UN’s Declaration of Human Rights (UDHR) principles [15], and in the case of health-related research involving human subjects, guidelines prepared by the Council for International Organization of Medical Sciences (CIOMS) [16]. For every ethical issue with questions, we developed the inference as the final judgement.

## Results

All public opinions expressed in the available media were considered and summarized including: statements, views and arguments directly or indirectly related to COVID-19 vaccine supply, policy of vaccination and the vaccine trial. A categorical description of the views concerning discussed issues, source of views and countries is provided in Table 1 and Table 2.

Most views in social media were expressed by public with just some examples of views provided by government officials and experts. The most frequently discussed issue was the quality of the vaccines offered by China or Russia as well as capacities for their local production. Governmental sources declared high probability of use vaccines from these countries for the mass vaccination. The public view also concentrated on choice between countries rather than manufacturers, with some touch of evidence issue. Another issue of discussion was mandatory vs. voluntary strategies with stressing possible rights violations at the first one.

“For whom you do this, for the people or for your own or political interest…the people should have a choice: who wants Chinese please, who wants Russian please…”

(middle-age man Uzbekistan)

“Putin himself advertised this, after vaccination re-infection is possible, but the disease proceeds in a mild form without complications and without health consequences”

(undefined user Kyrgyzstan)

“The problem is precisely the quaiity of the vaccine”

(middle-age man Kazakhstan)

“No one can tell me what medicine to inject me”

(young man, Uzbekistan)

A critical issue was the policy of recruiting subjects for COVID-19 vaccine trials. There was much public attention when the government in Uzbekistan provided a site for a phase III trial of the Anhui Zhifei Longcom Biopharmaceutical’s (China) vaccine. The Uzbek officials had declared that participation of 5,000 subjects in the trial would be voluntary, based on their formal consent. The authorized ministry of Uzbekistan stressed that its workers’ family members are also taking part in this trial as subjects. Nevertheless, a plurality of those commenting on these trials in the media claimed that Chinese vaccine producers may want Uzbek people to be “guinea pigs” for their trial. They held that Uzbek people should get only vaccines with proved effectiveness and safety. Only some postings accepted that there is nothing bad in taking part in such trial, especially if it results in an early vaccine supply.

“… and the Chinese one has not been tested on anyone at all, and our people are worse than monkeys to test on it? And at the expense of yourself prick yourself, so you bent it! I don’t need any! All is made (Russian slang) in an emergency mode, it is still unknown how later this vaccine will come back!”

(young woman Ferghana, Uzbekistan)

“Are Uzbek people rabbits to be tested by Chinese and Russians? Let them stupid, who signed such agreement, to take part on such testing. Do not touch ordinary people”

(middle-age woman Uzbekistan)

“Let the Uzbek vaccine be used exclusively for officials…”

(young man, Uzbekistan)

Postings by politicians and experts across the three countries predominantly supported the views of their governments. Public views remained evenly divided regarding their respective government policies and ranged from strongly negative to neutral or slightly positive.

### Ethical issues

The principal ethical issues arising in discussions within three countries over strategies to obtain COVID-19 vaccines arise in two categories. Ethical concerns included the acceptability of putting their citizens at possible risk so that the population can gain access to the vaccines it needs; and ethical issues in the conduct of the trial.

#### Ethical issue #1:

The Central Asian countries seek an inexpensive and easily-administered strategy to provide safe and effective COVID-19 vaccines for their communities, as soon as possible. The COVAX program was designed to provide vaccines that proved their quality in phase III trials and have been approved by WHO, but thus far this source of vaccines can meet at most 20% of what is needed. For the remainder, countries are on their own; and this is when the ethical choices arise. Their governments declared plans to import vaccines from Russia or China and even produce them internally. However, an efficacy and safety of COVID-19 vaccines from specific manufacturers may not be enough, so can harm countries’ communities. To provide faster benefit for more people, governments can choose mandatory vs. voluntary policy and this will become another ethical choice.

##### Questions.

- What kind of vaccine coverage and allocation strategy could be justifiable to provide access on highly restricted supply condition?
- Is this justifiable to import COVID-19 vaccines only from Russia or China and produce them locally despite of high concerns of these vaccines’ safety and efficacy?
- Is mandatory vaccination campaign justifiable, considering possibility of low efficacy in used vaccines and significant human rights violations?

#### Ethical issue #2:

A provision of subjects for the Zhifei Longcom’s vaccine phase III trial leaded to a privileged access to its COVID-19 vaccine for Uzbekistan. This guaranteed faster supply of the vaccine for more people in the country. However, the vaccine being tested can do harm in short or long term to subjects of the trial, even leading to their death. And its clinical efficacy possibly will not outweigh its potential risk. A level of ethical expertise, in the country, raise view about the high risk of misconduct during the trial and violation the rights of its subjects.

##### Questions.

- Is it acceptable to provide subjects for testing Chinese Zhifei Longcom’s vaccine to guarantee faster benefit for the subjects of the trial and all community in Uzbekistan?
- To what extent ethical requirements to protect human subjects are being followed in the trial conducted in Uzbekistan?

## Discussion

A developing strategy for success in obtaining and allocating COVID-19 vaccines as well as testing them becomes not easy task. This is, to our knowledge, the first study that analyzed public opinion regarding such strategies in low- and middle-income countries. We offered evidence of the debate within three Central Asian countries, drawn from social media postings and other sources. This debate caused ethical choices over which there may be considerable disagreement within each country. We are providing possible solutions for these choices with making practical inferences.

### Ethical issue #1

There is lack of reliable data from Central Asian countries concerning the herd immunity to SARS-CoV-2 in their communities. Data on other coronaviruses suggest that immunity might be short lived; however, goes to 12–18 months in duration [17]. Whether past infection prevents severe COVID-19 is still not clear. However, vaccines can sharpen immunity in previously infected people and contribute to mild illnesses in non-infected ones [18]. A spread of mutant strains that are more contagious and fatal [19] may contribute to reducing lockdowns effects and to the rise a role of the vaccination in the epidemic control. Last of them, Delta variant, looks deadliest this time [20] and is reaching Central Asia. This proves an urgent need in mass campaigns for all three countries. Uzbekistan and Kyrgyzstan started their campaigns in April, and Kazakhstan declared its start even earlier. Up to July 2021, there were 105 candidate vaccines in clinical and 184 in preclinical development, and 22 were within WHO evaluation process [21,22]. And an assessment to use for vaccination was finalized for seven vaccines, with no Russian vaccines in that list. However, the formal report about phase III trial of Sputnik V declared its efficacy at 91 % [23]. Until now, only Sinopharm from China got WHO approval for emergency use with 79% preliminary efficacy for symptomatic cases [24].

For countries with a larger population, such as Uzbekistan, low efficacy can make higher negative impact. An estimated threshold for COVID-19 vaccine efficacy, in case of full coverage, is about 60% at *R*_*0*_ equal to 2.5–3.5 [25]. The efficacy threshold rises to 70% when coverage drops to 34 and rises to 80% when coverage drops to 35 of the community. This makes countries more interested in a higher vaccination coverage in the case of lower vaccine efficacy. The most difficult is how to define target coverage in the case of variety of vaccines with different efficacy. By this time, the lowest declared efficacy rate (70%), among known candidates, is in Oxford-AstraZeneca vaccine [26]. This means that countries need 34 vaccination coverage in case they use only such vaccine as sole intervention. If consider that other applied vaccines may have higher efficacy, this coverage rate would be enough for a maximum public benefit. The cost of Sputnik V per patient is about half of the cost of Pfizer/BioNTech vaccine [27]. Chinese vaccines are comparable in cost with those from Western producers. A good point of non-Western vaccines is that they don’t require subzero storage, so they are better suited for mass distribution in warmer climates. However, Oxford-AstraZeneca vaccine can also be stored in regular fridge temperature.

A vaccine allocation strategy in Central Asian countries, including selection of vaccines for every individual use, rises serious ethical issues. In round the world, government officials demonstrate their adherence to COVID-19 vaccination. This also looks like the way to demonstrate that they share individual risk and benefit with their communities, in times of unclear or insufficient evidence about vaccines. However, most of such examples come from developed countries in which officials and communities are supposed to be vaccinated with the same vaccines, leading the similar risk and benefit among them. In Central Asia, vaccines’ deficit and potentially higher range in their efficacy and safety can lead to unequal distribution of individual risk and benefit, with possibility of an abuse of power. A low transparency can contribute to unjust vaccine access, leading the violation Article 21 (2) of UDHR. Our data proves a low public trust to governments, and social media users claim the officials of three countries to demonstrate their preparedness to get vaccinated with vaccines planned to be used for mass campaigns, that is to share the risk. This view can go even further, especially in times when cases of side effects and deaths after vaccines’ use are going up. An important factor becomes whether access to emergency care will be equal for all in case of side effects. Communities cannot expect such justice if they experienced access highly depending on their social status. In case public do not see the response to these issues, this situation can lead a vaccine rejection or hesitancy in Central Asia, where communities historically were highly adherent to vaccination. An applying some prioritization strategies and policies that propose to vaccinate specific subcommunities such as the elderly, live-saving or social service staff and close contact individuals [28, 29] will probably fail in this course.

From this view, an employment of mandatory vaccination looks inappropriate, this time, in Central Asia. We considered four additional conditions to make this decision [30]. First, a lower proportion of older population and lower than in Europe and US fatality rates make threat for public health less grave, in three countries. Kyrgyzstan has highest case fatality (1.71 %) in a smaller population, and this can be due to the policy of registration of all unclear deaths as COVID-19, in the peak of the epidemic. However, estimates of Institute for Health Metrics and Evaluation consider Central Asia as region with the highest ratio of total COVID-19 deaths to reported deaths [31]. An issue can be that all three countries had relatively low coverage by PCR testing, due to its high cost [32]. This can be a factor both increasing and decreasing the true epidemics burden in the countries. Second, even phase III trials data, there are common both experts and public concerns that vaccine testing has been rushed, and they may not be as safe or effective as they are declared. And this issue relates more to Russian and Chinese vaccines. This is condition decreasing public and individual benefit and increasing individual risk of the vaccination in three countries. Third, a systematic review of observational studies confirmed effectiveness of social distancing and wearing mask in reducing COVID-19 transmission [33]. This means that they still have high potential as alternatives to vaccination, especially in communities where they were not widely applied. Finally, another problematic issue is how proportionate can be coercion in a case of mandatory vaccination. In addition to unequal individual risk distribution, one can expect that coercion will be too strict, leading to significant human rights violation and social restrictions.

#### Inference

The policy of the vaccine supply, oriented to its imports only from specific countries (Russia or China) or producers cannot be justifiable this time. The market is developing fast and new vaccines appear to be available for mass campaigns in Central Asian countries. Their governments should consider their efficacy rates as a primary factor to plan coverage and selection of vaccines. This means that countries are recommended to use vaccines with a minimum 70% of efficacy; in this case they will need to provide 34 coverage by vaccination. The countries can use vaccines with a lower cost, in case they have this or higher efficacy rates. The cost will also include expenses for storage and transportation of vaccines. Political decisions and system of vaccines distribution should be transparent and communities of three countries should be informed about the vaccines’ selection pattern and about the way how equal sharing of individual risks and benefits will be provided. To prevent inequality in access to vaccines having different efficacy and safety, we would recommend, for every individual use to make random selection from the list of vaccines available in the country.

### Ethical issue #2

As an emergency way to test their vaccines, all manufacturers conduct clinical trials round the world, especially considering countries with big populations and high COVID-19 morbidity. Only Uzbekistan, in Central Asia, provides subjects for phase III trial of the Zhifei Longcom’s vaccine. And this time, it becomes the main vaccine used for a mass campaign in Uzbekistan. However, under WHO evaluation process, it is still marked as in step of expression of interest. The Uzbek study is a component of the trial that is registered in ClinicalTrials.gov as international multicenter study (ID: NCT04646590) having totally 18 study locations and requiring an informed consent from voluntary subjects. This time, a previously planned Uzbek sample size of 5000 subjects tends to be doubled, presumably, due to the fall in COVID-19 morbidity in the country or other locations. This means the number of subjects at risk is also doubling, while individual benefit from this vaccine testing is very low for these subjects. The reason is that COVID-19 is most fatal for older people having chronic conditions and their risk factors [34]. However, the trial involves only healthy young and middle-aged subjects genuinely having a low risk of severe disease, in case it develops on them. An authorized ministry, in Uzbekistan, declares that tested vaccine is safe because it is recombinant and does not contain virus. It is true that this type of vaccines has reduced side effects [35]; however, its safety concerns not only viral target but also variety of other ingredients and by-products of manufacturing.

From this view, it looks unreasonable to provide site for the trial as a way to get better access to the vaccine for the subjects of the trial. Moreover, the country cannot guarantee participation a specific number of subjects on the trial. The country’s role should be limited to formal permission to recruit subjects. The most important is that the country should protect rights of the subjects irrespective of what public benefit is expected to get from the tested vaccine. There is no information about the text of informed consent and in what degree the process of its collection complies with Guidelines 9 and 10 of CIOMS. From some sources it becomes clear that there can be government military workers among participants, so they can be vulnerable from the view of considering their rights as subjects of the trial. According to officials, the Ethics Committee at Ministry of Health has approved this research in the country. They also declare that the study will be stopped in case adverse effects will go in 30% of the involved subjects. However, a big issue becomes the transparency of the committee’s work and in what degree it is independent in its decisions.

#### Inference

A potentially low individual benefit cannot outweigh the risk for subjects involved to Zhifei Longcom’s vaccine phase III trial, in Uzbekistan. The policy to provide site for testing any specific COVID-19 vaccine can be justified only in a case the trial guarantees the protection rights of every subject. The Ethics Committee at Ministry of Health should provide a careful monitoring of the study, and it should have a right to stop the trial in any step. The informed consent should be delivered properly to subjects, leading their free agreement to participate in the study. By this time, the clinical efficacy and safety of Zhifei Longcom’s vaccine is unclear even relating to Uzbek participants of the trial. These findings become a critical point for a decisión to keep using this vaccine in Uzbekistán and for plans to provide coverage level.

### Limitations

This ethical analysis is not based on exact representation of public opinions related to COVID-19 vaccine supply or participation in the vaccine testing trial, in Central Asia. The proportion of communities that do not use social media or has steady Internet access can be high and can vary among countries. The idea was to collect all possible range of attitudes, in three countries, to adjust it with the basic ethical principles in the process of reasoning. Specific issues and questions extracted and discussed in our analysis can be distinct from other countries, and this can limit generalizability of our analysis. However, issues concerning COVID-19 vaccine supply become principal for every country, this time. The epidemic’s patterns and curves can change unpredictably in three countries, leading to higher COVID-19 morbidity and fatality rates. This would increase the public risk with its higher benefit from mandatory vaccination. However, this way will not resolve problems rising from the unequal risk and benefit distribution during the mass campaign.

## Conclusions

Despite the limitations of our study, we believe that it would be a helpful example of solving ethical issues that can rise concerning COVID-19 vaccine supply round the world. All countries should weigh risk and benefit from specific policies and stay very sensitive to the possible harm. The public view, even not basing on strong knowledge and expertise, can be highly critical, helping to spot such issues. An ignoring this view can lead to major problems, which in turn, can become a serious obstacle for the vaccine coverage and epidemics’ control in the countries and regions.

## Figures and Tables

**Table 1:** Number and distribution of views concerning discussed issues

	Uzbekistan	Kazakhstan	Kyrgyz Republic	Total
Vaccine import	21	36	18	75
Manufacturing local vaccines	7	11	9	27
Mandatory vs. voluntary vaccination	13	22	19	54
Participation in the vaccine trial	22	9	7	38
Others	3	5	6	14

**Table 2:** Number and distribution of views concerning their sources

	Uzbekistan	Kazakhstan	Kyrgyz Republic	Total
Government officials and politicians	6	11	3	20
Experts	4	6	5	15
Public (non-experts):	68	54	51	173
male	36	28	23	87
female	21	23	20	64
undefined	11	3	8	22

## Abbreviations

CIOMS: Council for International Organization of Medical Sciences
COVID-19: CoronaVirus Disease 2019
COVAX: COVID-19 Vaccine Global Access
LMICs: Low- and Middle-Income Countries
SARS-CoV-2: Severe Acute Respiratory Syndrome Coronavirus 2
PCR: Polymerase Chain Reaction
UDHR: United Nations Declaration of Human Rights
WHO: World Health Organization
